# Preparation and Identification of the Novel Umami Peptides from Sea Cucumber Viscera Hydrolysate

**DOI:** 10.3390/foods15040673

**Published:** 2026-02-12

**Authors:** Xinmiao Ren, Yiling Zhong, Changyun Wang, Qingping Liang, Shuang Li, Rongqiang Chen, Dongyu Li, Changliang Zhu, Xiaodan Fu, Haijin Mou

**Affiliations:** 1 College of Food Science and Engineering, Ocean University of China, Qingdao 266404, China; m13992623179@163.com (X.R.); zhongyiling321@163.com (Y.Z.); preenyliang@126.com (Q.L.); 15140923590@163.com (S.L.); rongqiang_chen@163.com (R.C.); ldy1516@ouc.edu.cn (D.L.); chlzhu@163.com (C.Z.); 2 School of Medicine and Pharmacy, Ocean University of China, Qingdao 266003, China; changyun@ouc.edu.cn

**Keywords:** *Apostichopus japonicus*, enzymatic hydrolysis, peptidomics, in silico screening, T1R1/T1R3 receptor, electronic tongue, sodium reduction, by-product valorization

## Abstract

Sea cucumber viscera by-products are abundant but remain underutilized. Although the development of umami peptides from marine by-products has been well-reported, sea cucumber viscera have received less attention. In this study, an umami-rich hydrolysate was prepared from sea cucumber viscera through synergistic dual-enzyme hydrolysis. Under optimal conditions, the co-hydrolysis using Flavourzyme and aminopeptidase yielded extraction rates of 69.38% for solids, 67.29% for protein, and 66.96% for total sugar, and produced a 1.75-fold higher umami signal intensity (electronic tongue) than the single-enzyme (Flavourzyme) hydrolysate. The target umami fraction was enriched through sensory-guided separation combined with ultrafiltration and ion-exchange chromatography. Thirty-three umami peptides, predominantly derived from actin hydrolysis, were identified in this fraction via peptidomics and virtual screening. Based on docking simulations against the umami receptor T1R1/T1R3, two peptides (DFLDDGPG and SDTGNFGF) with the lowest docking scores were selected. The predictions revealed that two peptides bind to the T1R3 subunit via hydrogen bonds and π-related interactions. The umami-enhancing effect of peptide DFLDDGPG in salty systems was demonstrated by a trained panel (*n* = 10) across concentration ranges of 0.1–1.0 mg/mL peptide and 0.1–1.0% NaCl, with a positive correlation validated by RSM and ANOVA (*p* < 0.05). This study identified novel umami peptides from sea cucumber by-products as promising candidates for natural, low-sodium flavor enhancers.

## 1. Introduction

Umami peptides, considered as natural seasoning agents to replace commercial umami taste food additives such as monosodium glutamate (MSG) and seasoned chicken or mushroom essence, have good nutritional value and bioactivity, and are easily metabolized by the human body [1]. As flavor-active ingredients, umami peptides are derived from various food matrices, including marine products, edible fungi, traditional fermented foods, and plant-based products [2]. Research indicates that umami peptides are a class of oligopeptides with molecular weights (MW) typically below 2000 Da. A large number of di- and tri-peptides isolated from yeast extract and soy sauce have umami tastes [3]. Xu et al. [4] identified mushroom-derived umami peptides, which possess low taste thresholds below 15 mmol/L. Marine organisms contain higher levels of umami amino acids than those in terrestrial counterparts. Species such as seaweed, fish, shrimp, crab, oyster, sea urchin, clam, sardine, and scallop are all known for their distinctive umami taste [5]. From pufferfish, an umami-rich source, seven novel oligopeptides (300–3000 Da) were identified, characterized by low taste thresholds (0.12–0.24 mmol/L) that are significantly lower in contrast to peptides from terrestrial sources [6]. Therefore, marine organisms represent a promising reservoir for umami peptides.

The viscera (comprising the intestinal tube, the respiratory trees, and the male and female gonads), representing a significant portion (up to 50% of total weight) of sea cucumbers, is frequently discarded despite sharing a similar protein composition with the commercially valued body wall. The intestine and gonad, as main visceral components, are particularly rich in flavor-active amino acids, where glutamate and aspartate provide umami, and alanine and glycine contribute sweetness [7]. Mild enzymatic hydrolysis has been employed to convert proteins into specific amino acids or peptides, enhancing the umami taste of the product [8]. Comparative analysis of amino acid composition in protein hydrolysates from different sea cucumber tissues (body wall, flower (gonad), and internal organs) has been conducted [9]. Gonad contains the highest proportion of umami and sweet-tasting amino acids (52.43%), followed by the body wall (51.62%), and the internal organs (45.41%). Although sea cucumber viscera are rich in protein and umami-related amino acids, their potential as umami peptide precursors has received much less attention. Studies on bioactive peptides derived from sea cucumber by-products have mainly been focused on activities such as damaged tissue repairing [10], antitumor [11], and antioxidant [12]. Sun et al. reported the activity and mechanism of sea cucumber intestinal peptides in protecting and repairing gastric mucosa [13]. While Liang et al. screened an ACE inhibitory peptide (NSPGDSFP) that possessed umami characteristics, the study mainly evaluated the ACE inhibition activity of the targeted peptide [14]. Currently, an effective approach for isolating and identifying umami components from the complex visceral matrix remains scarce.

For identifying and screening characteristic peptides, peptidomics combined with machine learning prediction models has been widely adopted. For instance, Zhu et al. [15] identify two antifreeze peptides from shrimp by-product autolysates using nano-LC-MS/MS analysis. Several machine-learning-based tools have been used for screening bioactive peptides, such as AntiFlam (for anti-inflammatory activity), TastePeptides-Meta (for umami taste), and BIOPEP-UWM (a database for antimicrobial and ACE-inhibitory peptides, among others) [16,17]. Umami peptides are characterized by an enrichment of acidic amino acid residues, typically involving one or two residues from glutamate, glutamine, aspartate, or asparagine [18]. In addition, peptides contain polar amino acids such as serine, threonine, and glycine, with a common structural motif featuring an aspartate or glutamate at the *N*-terminus, followed by a spacer sequence and one or more hydrophobic residues (e.g., alanine, valine) [19]. The interaction between amino acid residues and the T1R1/T1R3 taste receptor enables the neural transmission of the umami signal to the brain, where it is perceived as taste [20]. The heterodimer T1R1/T1R3 is recognized as the primary receptor for umami signaling [21]. Therefore, molecular docking of peptides with the T1R1/T1R3 receptor has been extensively applied in umami peptide research.

While sea cucumber viscera are rich in protein and umami amino acids, scant attention has been paid to their potential as precursors for umami peptides. An integrated route combining dual-enzyme hydrolysis, peptidomics, and in silico screening could be a promising approach to discover umami peptides from viscera, with positive sensory validation serving as the success criterion. This study aimed to optimize the hydrolysis process for an umami-rich hydrolysate, screen for candidate peptides using peptidomics-based in silico screening, and validate their umami characteristics via sensory evaluation. In this study, a two-step enzymatic hydrolysis process was optimized to produce a sea cucumber viscera hydrolysate with pronounced umami taste. Subsequently, two novel umami peptides were screened via ion-exchange chromatography, LC-MS/MS-based identification, umami prediction, and molecular docking. The synergistic umami-enhancing effect of the two identified peptides with NaCl was investigated. This work provides a theoretical foundation for the high-value utilization of sea cucumber by-products and the development of novel, low-sodium condiments.

## 2. Materials and Methods

### 2.1. Materials

Frozen sea cucumber (*Apostichopus japonicus*) by-products (viscera) were supplied by Weihai Dipusen Biotechnology Co., Ltd. (Weihai, China). Neutrase, Flavourzyme, papain, aminopeptidase, Alcalase, and Subtilisin were obtained from Shandong Guoli Biotechnology Co., Ltd. (Jinan, China). Q-Sepharose Fast Flow anion-exchange resin was purchased from Cytiva (Uppsala, Sweden). LC-MS grade formic acid was supplied by Macklin (Shanghai, China). Acetonitrile was obtained from Fisher Chemical (Hampton, NH, USA). Urea was purchased from Aladdin (Shanghai, China). Peptides were synthesized by Sangon Biotech Co., Ltd. (Shanghai, China) with a purity exceeding 90%. Food-grade citric acid, sucrose, sodium chloride (NaCl), and MSG were obtained commercially. All other chemicals and solvents used were of analytical grade.

### 2.2. Double Enzyme Synergistic Hydrolysis of Sea Cucumber by-Products

#### 2.2.1. Enzyme Screening and Optimization of Enzymatic Hydrolysis Conditions

Sea cucumber viscera were subjected to hydrolysis. For the enzyme 1, Alcalase (pH 10.0, 40 °C), Neutrase (pH 7.0, 45 °C), Subtilisin (pH 7.5, 45 °C; 120,000 U/g), papain (pH 7.0, 50 °C), and Flavourzyme (pH 6.0, 50 °C) were screened. Hydrolysis was performed with an enzyme activity of 1000 U/g, a substrate concentration of 10% (*w*/*v*), for 6 h. The reaction was terminated by heating at 90 °C for 10 min, followed by centrifugation at 8000× *g* for 10 min to collect the supernatant. Solid yield, protein yield, and total sugar yield were determined using the constant-weight method, the Kjeldahl method, and the phenol-sulfuric acid method, respectively, as described by Phuong et al. [22]. Efficient protein and solid solubilization provides sufficient flavor precursors from the visceral matrix [8]. Using Flavourzyme as the enzyme 1, the effects of enzyme activity, substrate concentration, and reaction time on solid, protein, and total sugar yields were investigated to determine the optimal hydrolysis conditions.

#### 2.2.2. Establishment of Double Enzyme Synergistic Hydrolysis

Under the optimal conditions for Flavourzyme (1000 U/g, 10% substrate, pH 6.0, 50 °C, 20 h), it was combined with a second enzyme (neutral protease, papain, or aminopeptidase) to improve the flavor of hydrolyzed products. Based on preliminary experiments, a fixed enzyme activity of 500 U/g was applied to the screening process. Solid yield, protein yield, total sugar yield, and electronic tongue taste profiles were used to select enzyme 2. For electronic tongue analysis, the frozen hydrolysate was reconstituted in ultrapure water at 1 mg/mL to maintain conductivity within the instrument’s recommended operating range (1–10 mS/cm). Each sample was analyzed over four consecutive measurement cycles; the first cycle was discarded for sensor stabilization, and the mean of the remaining three cycles was used for data reporting. The relative standard deviation across replicate measurements was <5%. Taste profiles were analyzed using a SA402B electronic tongue system (Intelligent Sensor Technology, Inc.,Kanagawa, Japan) equipped with five dedicated sensors (AAE, CT0, CA0, C00, AE1) for quantifying umami, saltiness, sourness, bitterness, and astringency, respectively.

### 2.3. Separation and Purification of Umami Peptides Using Anion-Exchange Chromatography

The hydrolysate was dissolved in distilled water and fractionated using a 10 kDa MWCO TFF membrane cassette (Millipore, Billerica, MA, USA) with a digital constant-flow pump to remove residual enzymes and proteins in the hydrolysate. The fraction below 10 kDa was collected for further purification. The ultrafiltered sample was loaded onto a Q-Sepharose Fast Flow column (2.6 × 20 cm) pre-equilibrated with 20 mM sodium phosphate buffer (pH 7.0). For each run, 100 mg of peptide sample was loaded, dissolved in 5 mL of buffer to a concentration of 20 mg/mL. After the sample application, bound peptides were eluted with a step gradient of 0, 0.1, 0.2, and 0.4 M NaCl in the same buffer at a flow rate of 1 mL/min. Fractions (5 mL/tube) were collected manually during the isocratic elution at each salt concentration step, based on the UV absorbance profile at 280 nm. This chromatographic step was performed in triplicate (*n* = 3) with good reproducibility, and the corresponding fractions from multiple runs were pooled. Protein content was determined using a BCA assay. Four major pooled fractions (a–d) were desalted (100 Da MWCO dialysis), concentrated by rotary evaporation, lyophilized, and analyzed by electronic tongue as detailed in Section 2.2.2 to identify the fraction with the highest umami response for identification of peptides.

### 2.4. Umami Peptide Screening and Prediction Based on Peptidomics

Peptide identification was conducted as described previously [23] with some modifications. Fraction c was dissolved in mobile phase A (0.1% formic acid in water), filtered (0.22 μm), and analyzed by LC-MS/MS using an EASY-nanoLC 1200 system coupled to a Q-Exactive HF mass spectrometer (Thermo, Waltham, MA, USA). Separation was performed on an Acclaim PepMap RSLC C18 column (50 μm × 150 mm, 2 μm, Thermo) with the gradient elution procedure. Elution was solvent A (0.1% formic acid -water solution) and solvent B (80% acetonitrile and 0.1% formic acid–water solution). The elution program was as follows: 0–98 min: 8% to 28% B, 98–113 min: 28% to 37% B, 113–117 min: 37% to 100% B, 117–120 min: 100% B (hold). A constant flow rate of 0.30 μL/min was maintained throughout the analysis, with an injection volume of 1 μL. MS settings: positive ion mode; full scan range 400–1800 m/z at 60,000 resolution; data-dependent acquisition of the top 20 precursors; HCD collision energy 28 eV. For polypeptide identification, raw files were processed via the database search (uniprot-taxonomy_7684) using Proteome Discoverer version 2.5 (Thermo, Waltham, MA, USA). The parameters used were: no-enzyme (unspecific), carbamidomethyl (C) of the fixed modifications, and oxidation (M), acetyl/+42.011 Da (N-terminus), Met-loss/–131.040 Da (M) (N-terminus), and Met-loss + acetyl/–89.030 Da (M) (N-terminus) of the variable modifications, 10 ppm of the peptide mass tolerance, 0.02 Da of the fragment mass tolerance, and PSM/peptide level FDR ≤ 0.01. Peptides identified by LC-MS/MS were filtered for a molecular weight range of 400–1000 Da. Umami potential was predicted using two machine-learning tools, UMPred-FRL (http://pmlabstack.pythonanywhere.com/UMPred-FRL accessed on 8 June 2025) and TastePeptides-DM (http://www.tastepeptides-meta.com accessed on 8 June 2025), with a prediction score threshold of >0.5. UMPred-FRL is a dedicated umami predictor, while TastePeptides-DM employs a binary umami/bitter classification model. Candidate peptides were further evaluated for bioactivity probability (>0.5) using PeptideRanker (http://distilldeep.ucd.ie/PeptideRanker/ accessed on 9 June 2025). Finally, toxicity was predicted using the ToxinPred 3.0 (https://webs.iiitd.edu.in/raghava/toxinpred3 accessed on 9 June 2025).

### 2.5. Molecular Docking of Umami Peptides with T1R1/T1R3

The molecular docking simulation was conducted according to previous studies [24,25]. The 3D structure of the ligand-binding domain of the human T1R1/T1R3 heterodimer (UniProt IDs: Q7RTX1, Q7RTX0) was constructed using the SWISS-MODEL server (https://swissmodel.expasy.org/ accessed on 16 June 2025). The homology model of the T1R1/T1R3 receptor in this study was constructed using the cryo-EM structure of mGlu2-mGlu3 as the template (SMTL ID: 8wjc.1, corresponding to PDB ID: 8WJC). The sequence identity between the target T1R1/T1R3 protein and this template was 71.62%. Model quality was validated with SAVES v6.0; a Ramachandran plot confirming >90% of residues in favored/allowed regions was required for model acceptance [26]. Peptide structures were drawn in ChemDraw 19.0 and converted to 3D conformations in Chem3D 19.0. Semi-flexible docking was performed using PyRx software (version 0.9.4). The grid box was centered on the active site of the T1R1/T1R3 receptor. The grid size = 66 × 98 × 160 Å. The exhaustiveness parameter was set to 8, and the number of runs was set to 50. The resulting complexes with the lowest binding free energies were selected for detailed analysis. Interaction modes, binding sites, and intermolecular forces were visualized and analyzed using PyMOL (v2.5.5) and Discovery Studio (version 2019) . The two peptides identified were input to pLM4Alg (https://f6wxpfd3sh.us-east-1.awsapprunner.com/ accessed on 24 June 2025) for potential allergenicity prediction [27].

### 2.6. Solid-Phase Synthesis and Sensory Evaluation of Two Umami Peptides

The two peptides with the lowest docking free energies were selected for synthesis. They were prepared by solid-phase synthesis, followed by desalting, by Sangon Biotech Co., Ltd. (Shanghai, China), with a final purity exceeding 90%. Sensory evaluation was conducted with reference to the method described by Chen et al. [28], with minor modifications. Participants aged 20–40 years were recruited and screened (non-smokers, no spicy food before sessions, no taste/olfactory disorders). Their discrimination of the five basic tastes was evaluated by correctly ranking five concentration gradients of each: salty (NaCl: 0.10–0.50%), sweet (sucrose: 1.0–10.0%), umami (MSG: 0.10–0.50%), bitter (caffeine: 0.02–0.15%), and sour (citric acid: 0.02–0.20%). Only those who passed proceeded to umami-specific training. Using three MSG reference solutions (1, 4, 8 mmol/L, corresponding to scores of 1, 5, and 10), panelists learned to differentiate intensities and then matched unknown samples to these references. Ten qualified panelists (5 male, 5 female) were finally selected. Ethical approval was obtained prior to the study.

The synthetic peptides were dissolved in ultrapure water to prepare solutions at concentrations of 0, 0.5, and 1 mg/mL. Each peptide concentration was further supplemented with NaCl at 0, 1.5, and 3 mg/mL, establishing a two-factor, three-level experimental design for response surface methodology (RSM). The central composite design comprised 10 trained panelists. Each experimental point was evaluated in triplicate. To assess the repeatability of the sensory procedure, the entire evaluation was repeated in an independent session one week after the initial assessment. The lack of a significant difference (*p* > 0.05) between the geometric mean intensity scores of the two sessions confirms the high repeatability of our sensory method. Samples were presented in a single-blind, randomized order using three-digit codes to minimize bias. Peptide concentration (A) and NaCl concentration (B) were the independent variables, and the mean umami intensity score (Y) was the response variable. In the statistical analysis of the RSM model, assessor variability was accounted for by including Assessor as a random effect in the ANOVA. Design-Expert software (version 13) was used to generate the response surface plots and the equations for predicting the response variable.

### 2.7. Statistical Analysis

All data was presented as mean and standard deviation (SD). The data analysis of variance (ANOVA) and Duncan’s test were performed on pairs using SPSS 26.0 software (SPSS, Inc., Chicago, IL, USA). Results were considered statistically significant at *p* < 0.05. Origin Pro 2021 (OriginLab Corp., Northampton, MA, USA) was employed for data visualization.

## 3. Results and Discussion

### 3.1. Process Optimization of Double Enzyme Synergistic Hydrolysis for Sea Cucumber by-Products

**Figure 1:**
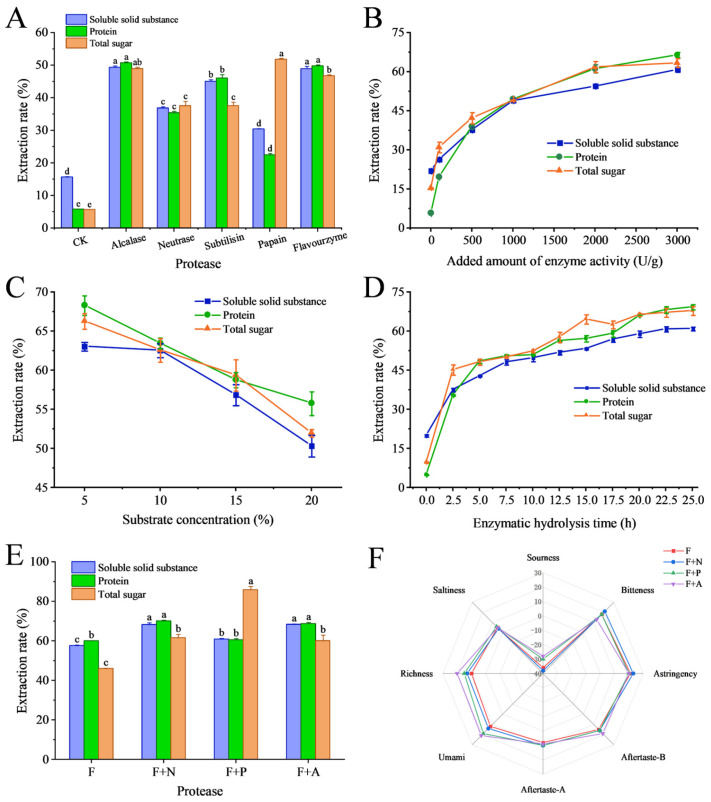
Enzyme screening and optimization of enzymatic hydrolysis conditions. Effect of protease type (**A**), amount of enzyme activity (**B**), substrate concentration (**C**), and hydrolysis time (**D**) on extraction rates of solids, protein, and total sugar. Effect of complex enzyme type on the extraction rate of solids, proteins, and total sugars (**E**), and the effect on taste (**F**). F: Flavourzyme, N: Neutrase, P: papain, A: aminopeptidase. Different letters indicate significant differences as determined by one-way ANOVA followed by Tukey’s post hoc test (*p* < 0.05).

Enzymatic hydrolysis is a mild and effective bioprocessing technique widely employed in the processing of aquatic by-products [29]. Given the variation in hydrolysis efficiency among different enzymes when applied to sea cucumber by-products, optimizing the reaction conditions is crucial for enhancing both the yield and quality of the resultant hydrolysate [30]. In this study, sea cucumber viscera were subjected to hydrolysis, and the yield of solid, protein, and total sugar was used as a key parameter to screen candidate enzymes. Among the enzymes tested, alkaline protease achieved the highest solid and protein yields, followed by Flavourzyme (Figure 1A and Table S1). Both enzymes also showed high total sugar yields, second only to papain. A critical drawback of alkaline protease is its optimal operating pH of 9.5–10.5. Consequently, post-hydrolysis neutralization with HCl is required, which introduces salts and necessitates a costly desalting step for food-grade production. In contrast, Flavourzyme, with an optimal pH of 6.0, eliminates the need for both neutralization and desalting. Therefore, based on a balanced consideration of yield, cost-effectiveness, and process simplicity, Flavourzyme was selected as Enzyme 1.

To determine the optimal working conditions for Flavourzyme, the effects of enzyme activity, substrate concentration, and reaction time were investigated. As shown in Figure 1B, solid, protein, and total sugar yields increased rapidly as enzyme activity rose from 0 to 1000 U/g, indicating enhanced substrate-enzyme interaction and accelerated release of intracellular materials. Beyond 1000 U/g, the increase in yields slowed, suggesting saturation of available binding sites between enzyme and substrate. Substrate concentration was then varied (5%, 10%, 15%, and 20% (*w*/*v*)) at a fixed enzyme activity of 1000 U/g. Yields of solids, protein, and total sugar decreased as substrate concentration increased (Figure 1C), likely due to increased viscosity and reduced mass transfer limiting enzyme-substrate contact. The decline was moderate between 5% and 10% but became pronounced above 10%, establishing 10% as the optimal substrate concentration. Reaction time was optimized under the determined conditions (1000 U/g, 10% substrate, pH 6.0) over 25 h period. Yields increased rapidly within the first 2.5 h, continued to rise at a slower rate until 20 h, and plateaued thereafter (Figure 1D). This trend reflected sustained enzymatic activity followed by a gradual completion of material release. Consequently, 20 h was selected as the optimal hydrolysis time.

Despite achieving yields over 50% under the optimal condition (1000 U/g, 10% substrate, pH 6.0, 20 h), the hydrolysate produced by Flavourzyme alone required sensory improvement of the sensory properties. The bitter taste common in protein hydrolysates is primarily attributed to the exposure of hydrophobic amino acid moieties during hydrolysis [31]. To improve the flavor profile of the hydrolysate, a secondary enzyme (Complex Enzyme 2) was selected to complement Flavourzyme. Electronic tongue analysis indicated that combining Flavourzyme with aminopeptidase significantly enhanced umami and richness, while pairing it with neutral protease intensified bitterness, likely from the generation of small, bitter peptides (Figure 1F). Aminopeptidase, an exoprotease, hydrolyzes peptide bonds at the N-terminus, releasing free amino acids that can improve flavor and reduce bitterness [32]. Although the Flavourzyme-aminopeptidase combination showed lower solid and protein yields than the Flavourzyme-Neutrase pair (Figure 1E), aminopeptidase was selected for the second hydrolytic step as Enzyme 2 to generate umami-tasting hydrolysates. After hydrolysis with Flavourzyme (1000 U/g) and aminopeptidase (500 U/g) at 50 °C for 20 h (10% substrate, pH 6.0), extraction yields of 69.38% (solids), 67.29% (protein), and 66.96% (total sugar) were achieved, along with a 1.75-fold higher umami signal intensity relative to that produced by Flavourzyme alone.

### 3.2. Separation and Sensory Assessment of Umami Peptides

**Figure 2:**
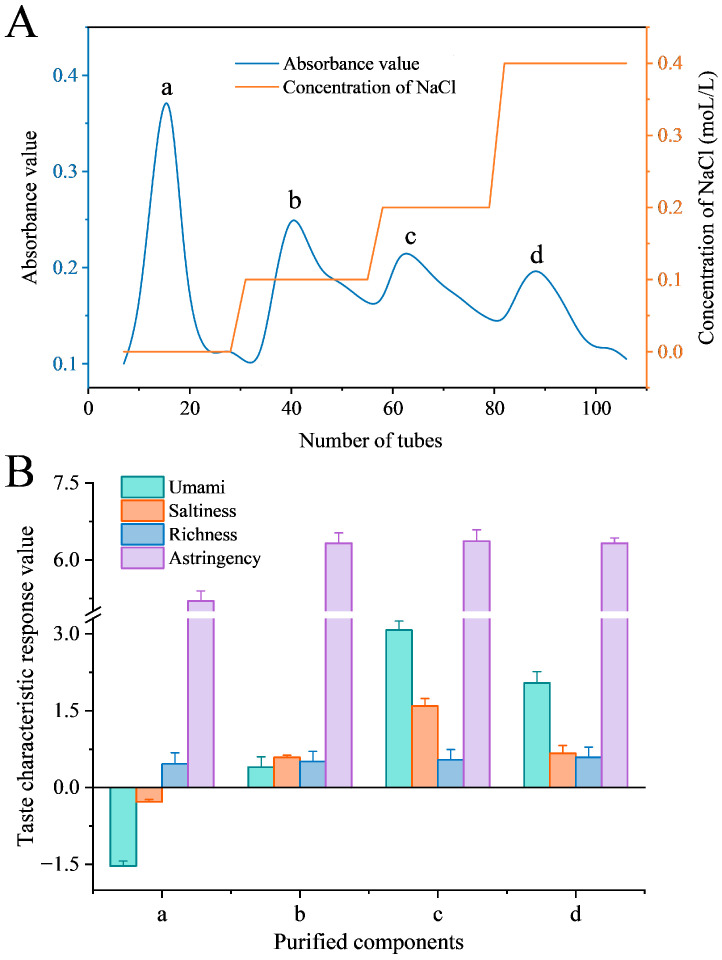
Anion exchange chromatography of sea cucumber viscera hydrolysate and taste characteristics of the four fractions. (**A**) Purified components of SCE obtained on Q-FF. (**B**) The taste characteristic response values of each purified component.

Umami peptides are typically rich in glutamate and aspartate, which confer a net negative charge at physiological pH [33]. Studies indicate that a higher content of these acidic amino acids generally correlates with a stronger umami taste [26]. Based on charge property, a strong anion-exchange column was employed to purify umami peptides from the hydrolysate via electrostatic adsorption. To achieve precise separation based on ionic strength, a stepwise NaCl gradient (0, 0.1, 0.2, and 0.4 mol/L in phosphate buffer) was applied, resolving the mixture into four major fractions (fractions a–d; Figure 2A). The taste profiles of these fractions were evaluated using an electronic tongue (Figure 2B). Fraction a produced a negative umami response, indicating no umami taste. Fraction b showed a low response (0.40). The highest umami intensity was recorded for fraction c (3.07), followed by fraction d (2.04). The increasing umami of fractions c and d compared to fractions a and b aligned with the principle that a stronger ionic strength is required to displace more strongly bound, highly negatively charged peptides. However, the chromatographic elution behavior of umami peptides is determined by the combined effects of multiple physicochemical properties, including hydrophobicity, charge, spatial structure, and receptor-specific affinity [5,34]. The peak umami response was found in fraction c, not in the final fraction d eluted at the highest salt concentration. In addition, saltiness and astringency responses followed a trend similar to umami, also peaking in fraction c. The “richness” attribute was highest in fraction d, which is consistent with theoretical expectations. However, no significant difference was observed between fractions c and d. In contrast, the umami intensity of fraction c was significantly higher than that of fraction d (*p* < 0.05). Kokumi refers to a composite sensory attribute associated with richness, mouthfulness, and long-lasting complexity, whereas umami is recognized as a distinct, basic taste. Therefore, fraction c with the highest umami signal intensity was collected as the target fraction for peptide identification [35]. Therefore, fraction c was collected as the target fraction for subsequent peptide identification.

### 3.3. Screening and Characterization of Umami Peptides

**Figure 3:**
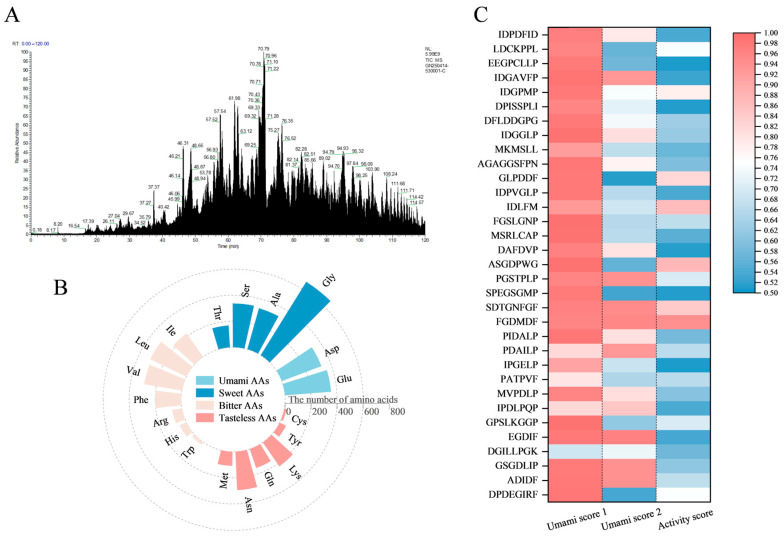
Peptidome analysis of umami fraction c. (**A**) Total ion chromatogram of component c obtained by LC-MS/MS. (**B**) Amino acid composition of peptides with molecular weight distribution in 400–1000 Da. (**C**) Heat map analysis of umami and activity scores of candidate peptides.

The peptide sequences in fraction c were identified by LC-MS/MS (Figure 3A). The precursor proteins of these peptides were analyzed to pinpoint the source proteins of umami peptides in the sea cucumber by-products. The precursor proteins were predominantly mapped to actin, which exhibited the highest peptide coverage (>50%). Actin serves as a core structural component of the eukaryotic cytoskeleton and is notable for its high intracellular abundance [36]. Most umami peptides are derived from the hydrolysis of myofibrillar proteins by endogenous enzymes or microorganisms [23]. Bai et al. [37] reported that lactic acid bacteria promoted the degradation of myofibrillar protein into umami-enhancing peptides. Specifically, five umami-enhancing peptides identified in that study were all derived from myofibrillar protein in dry-cured mackerel.

Previous research indicates that umami peptides with a MW below 1500 Da possess high umami taste [38]. Among marine-derived umami peptides, short-chain peptides (di- to heptapeptides) represent a predominant fraction, accounting for 54.56% of the distribution [19]. Peptide chain length is a critical factor affecting umami intensity, with the strongest umami taste generally associated with tetra- to hexapeptides (4–6 amino acid residues) [39,40]. Therefore, our study focused on medium-length peptides within the 400–1000 Da range, which accounted for the most abundant fraction (67.82%) of the peptide pool. A total of 670 peptides within the target MW range were identified, and the amino acid composition is shown in Figure 3B. The most abundant amino acids were glycine (Gly, 15.76%), glutamic acid (Glu, 8.45%), alanine (Ala, 7.78%), and aspartic acid (Asp, 7.74%), predominantly consisting of umami/acidic amino acids and hydrophobic amino acids. Collectively, the main taste profile of the peptide set was characterized by sweet-tasting amino acids (35.56%) and umami-imparting amino acids (16.19%). To screen for umami potential, the 670 peptides were subjected to prediction using two machine-learning-based tools (UMPred-FRL and TastePeptides-DM). From this analysis, 33 candidate peptides with a prediction score > 0.5 were selected (Figure 3C). The bioactive potential of these 33 candidates was further assessed using PeptideRanker, a tool that ranks peptide sequences by their probability of exhibiting bioactivity. All 33 peptides were assigned a bioactivity probability > 0.5. Finally, the toxicity of all candidates was predicted using ToxinPred, a classifier trained on toxic and non-toxic peptide data. All 33 candidate peptides were predicted to be non-toxic.

### 3.4. Molecular Docking of Umami Peptides with the T1R1/T1R3 Receptor

To explore the interaction between the identified peptides and the umami taste receptor, a homology-modeled 3D structure of the human T1R1/T1R3 heterodimer was constructed (Figure S1A–C). The T1R1/T1R3 receptor has two large N-terminal extracellular domains with a bilobed structure called the Venus flytrap domain (VFTD) [41]. Various taste-active peptides have been reported to interact with the T1R1/T1R3 subunits [26,34]. Analysis of the Ramachandran plot validated the model’s reliability, showing that 93.0% of amino acid residues were located in the most favored regions, with an additional 7.0% in allowed regions (Figure S1D). The 3D structures of the 33 candidate umami peptides were subjected to geometry optimization and energy minimization to obtain stable low-energy conformations suitable for docking. Molecular docking was performed to predict the potential interactions between 33 umami peptides and the T1R1/T1R3 receptor. All peptides yielded negative docking energies (Table 1), where a lower docking score correlates with a stronger affinity and a more stable complex [42]. Among the 33 peptides screened, DFLDDGPG exhibited the lowest docking score (docking energy: −7.7 kcal/mol), followed by SDTGNFGF at −7.6 kcal/mol. There was an obvious correlation between the docking score and the acidic amino acid (Asp/D, Glu/E), which were generally the major components of umami peptides [40]. The docking analysis showed that hydrogen bonds and π-related interactions occurred between the two umami peptides and T1R1/T1R3.

**Figure 4:**
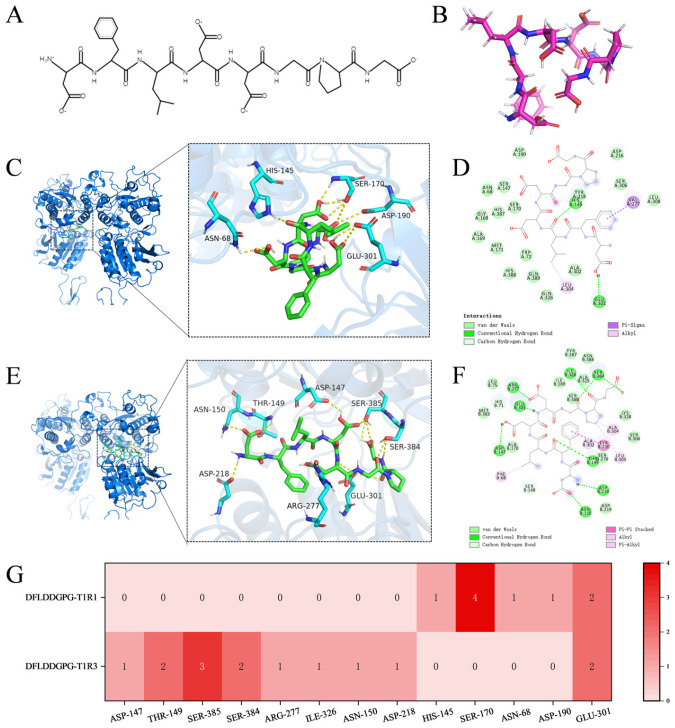
Molecular docking analysis of peptides DFLDDGPG with umami receptors T1R1/T1R3. (**A**) 2D structural representation of DFLDDGPG; (**B**) 3D conformational model of DFLDDGPG; (**C**) 3D binding pose of DFLDDGPG with T1R1; (**D**) 2D interaction diagram of DFLDDGPG with T1R1; (**E**) 3D binding pose of DFLDDGPG with T1R3; (**F**) 2D interaction diagram of DFLDDGPG with T1R3; (**G**) Heatmap analysis of key binding residues between DFLDDGPG and T1R1/T1R3.

**Figure 5:**
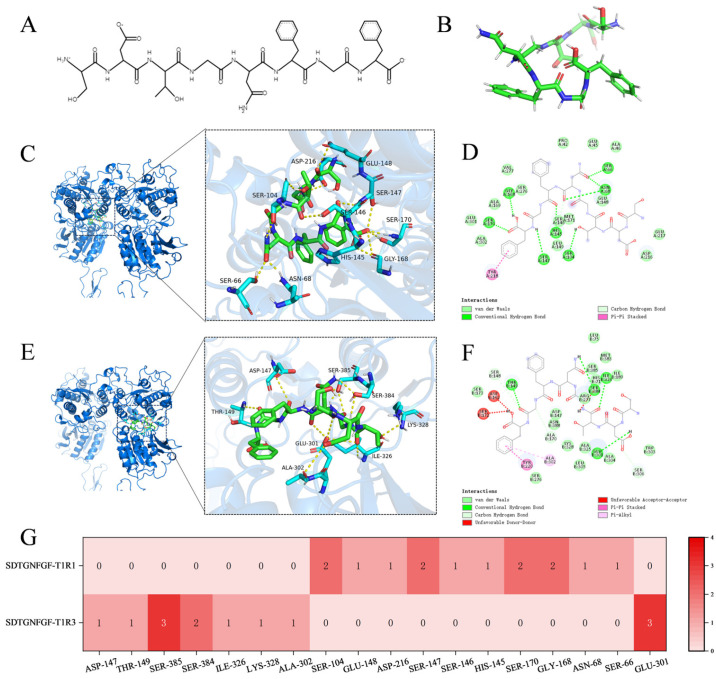
Molecular docking analysis of peptides SDTGNFGF with umami receptors T1R1/T1R3. (**A**) 2D structural representation of SDTGNFGF; (**B**) 3D conformational model of SDTGNFGF; (**C**) 3D binding pose of SDTGNFGF with T1R1; (**D**) 2D interaction diagram of SDTGNFGF with T1R1; (**E**) 3D binding pose of SDTGNFGF with T1R3; (**F**) 2D interaction diagram of SDTGNFGF with T1R3; (**G**) Heatmap analysis of key binding residues between SDTGNFGF and T1R1/T1R3.

DFLDDGPG, an octapeptide featuring a phenyl ring (Figure 4A,B), was predicted to interact with T1R1 primarily through residues SER-170 and GLU-301, with interactions dominated by van der Waals forces and two hydrogen bonds (Figure 4C,D,G). In contrast, its binding to T1R3 involved a more extensive network, including key residues SER-385, THR-149, SER-384, and GLU-301. This interaction was supported by a total of eight hydrogen bonds and was further characterized by π–π stacking between the peptide’s phenyl ring and aromatic residues in the receptor (Figure 4E–G). The broader and more complementary binding interface of T1R3 contributed to a more favorable docking score compared with T1R1. Docking predictions for SDTGNFGF, an octapeptide containing two phenyl rings (Figure 5A,B), indicated distinct binding modes for two receptor subunits. In T1R1, seven hydrogen bonds (involving SER-104, SER-147, SER-170, and GLY-168) and π–π stacking were observed (Figure 5C,D,G). For T1R3, the predicted interaction centered on a coordinated polar surface by SER-385, SER-384, and GLU-301, facilitating four hydrogen bonds alongside π–π and alkyl–π interactions (Figure 5E–G). Although more hydrogen bonds were predicted with T1R1, the specific and synergistic geometry of the interactions within the T1R3 binding pocket, coupled with the conformational constraints imposed by the peptide’s rigid structure, corresponded to a lower docking score with T1R3.

DFLDDGPG and SDTGNFGF exhibited the lowest docking scores (−7.7 and −7.6 kcal/mol, respectively) and the highest umami prediction probability (1.0). Both peptides contain characteristic amino acid residues of umami peptides, including acidic, aromatic, and hydrophobic types, representing a common compositional feature among all 33 predicted candidates. In addition, the two peptides were assessed for potential allergenicity using the pLM4Alg, and both were predicted to be non-allergenic. The molecular docking analysis indicates potential interaction patterns. To further explore the umami mechanism, future studies could incorporate molecular dynamics simulations and cell-based receptor assays.

### 3.5. The Umami-Enhancing Effect of Two Umami Peptides

**Figure 6:**
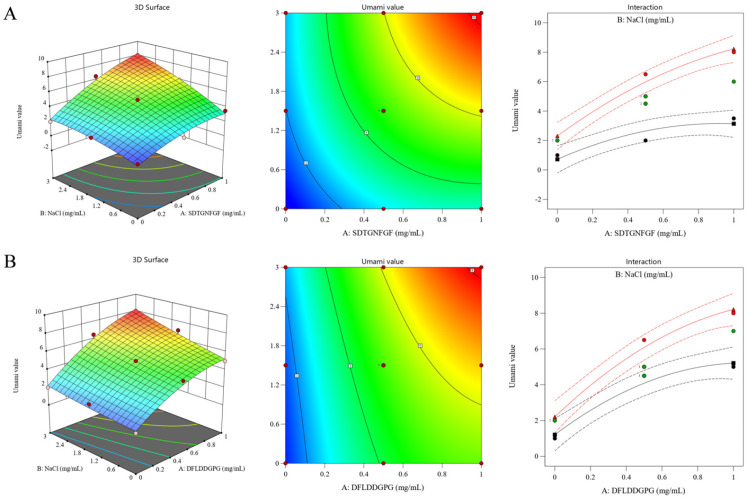
Synergistic enhancement of umami taste by SDTGNFGF (**A**) and DFLDDGPG (**B**) in the presence of NaCl. In the interaction plots (right), red, green, and black points represent experimental data at high, central, and low levels of NaCl, respectively.

To investigate the umami-enhancing properties of DFLDDGPG and SDTGNFGF, response surface methodology (RSM) was applied to the sensory analysis, which has been used to study the interaction of umami and NaCl in salt reduction [43]. NaCl was employed as the background medium in the sensory evaluation. The synergistic effect of peptide and NaCl concentration on perceived umami intensity was modeled and visualized with 3D response surface plots (Figure 6). NaCl lowers the resting potential of taste receptor cells, increasing their sensitivity to stimuli, while also raising solution viscosity to extend tastant-receptor contact time [44]. In the sensory framework, saltiness acts as a foundational matrix, which is beneficial for the clearer perception of the umami signal.

Both peptides showed a strong umami taste in the presence of NaCl. Umami intensity was positively correlated with increases in both peptide and salt concentration. Umami intensity increased rapidly at peptide concentrations below 0.5 mg/mL, followed by a more gradual increase between 0.5 and 1.0 mg/mL. Distinct patterns were observed for each peptide. High umami intensity for SDTGNFGF (0.8–1.0 mg/mL) required a correspondingly higher NaCl concentration (2.4–3.0 mg/mL). In contrast, DFLDDGPG at the same peptide concentration achieved high umami levels at a lower salt concentration (1.2–1.8 mg/mL). The data for SDTGNFGF and DFLDDGPG were fitted to obtain the following binary quadratic regression equations:

(1) SDTGNFGF: Y = 4.78 + 2.08A + 1.67B + 0.8750AB − 0.7155A^2^ + 0.4655B^2^ (R^2^ = 0.9729)

(2) DFLDDGPG: Y = 4.97 + 2.5A + B + 0.5AB − 0.8793A^2^ + 0.1207B^2^ (R^2^ = 0.9731)

where A represented the coded peptide concentration and B represents the coded NaCl concentration.

Analysis of variance (ANOVA) indicated that both models were highly significant (*p* < 0.0001), with non-significant lack-of-fit terms (*p* > 0.05). The R^2^ values exceeding 0.95 demonstrate a strong correlation between the measured and predicted values in this two-factor, three-level experimental design. While this study supported the concentration-dependent synergistic umami enhancement between the identified peptides and NaCl, the effect size relative to commercial enhancers such as MSG or nucleotides (IMP/GMP) remains to be quantified.

The observed umami-enhancing effect of peptides DFLDDGPG and SDTGNFGF in the NaCl-containing sensory model suggests their potential stability and functional compatibility in common salty food matrices, such as soups and sauces. However, the stability of novel umami peptides under specific food processing conditions requires systematic evaluation. Furthermore, producing umami peptides from sea cucumber viscera, a low-value processing by-product, represents a cost-effective production strategy [45]. Derived from an edible marine source through food-grade enzymatic hydrolysis, these peptides serve as potential candidates for natural flavor enhancers. For future industrial applications, establishing mature, scaled-up processes to address off-flavors, contaminants, batch variability, and production cost per kilogram is essential. Beyond production, the applicational performance of peptides must be validated, including their recognition thresholds in complex food matrices, chemical stability during processing and storage (shelf life), and their cross-modal sensory impacts [7].

## 4. Conclusions

In this study, sea cucumber viscera, a major processing by-product, were hydrolyzed via a dual-enzyme synergistic process to explore novel umami peptides. Under optimized conditions using Flavourzyme (1000 U/g) and aminopeptidase (500 U/g) at 50 °C for 20 h (10% substrate, pH 6.0), high extraction yields were obtained for solids (69.38%), protein (67.29%), and total sugar (66.96%). The dual-enzyme hydrolysate showed a 1.75-fold higher umami signal than the Flavourzyme-only control. Anion-exchange chromatography isolated an umami-rich fraction, which showed the strongest umami, salty, and astringent responses among the four separated fractions. Peptidomic analysis (LC-MS/MS) of the fraction identified 670 peptides within the 400–1000 Da range. Subsequent screening with UMPred-FRL and TastePeptides-Meta identified 33 candidate umami peptides, all of which were predicted to bind to the human T1R1/T1R3 receptor in molecular docking. Among them, two octapeptides, DFLDDGPG and SDTGNFGF, exhibited the lowest binding energies (−7.7 and −7.6 kcal/mol, respectively) and demonstrated a concentration-dependent synergistic umami enhancement in the presence of NaCl. This study provides an effective strategy for developing novel, natural umami ingredients from sea cucumber viscera, promoting higher-value utilization of this processing by-product. The candidate peptides were identified using a combination of in silico simulation, electronic tongue analysis, and sensory evaluation. The specific mechanism of the umami peptides and the processes toward cost-effective and large-scale application require future exploration.

## Figures and Tables

**Table 1:** The docking free energy of umami peptides with T1R1/T1R3.

Number	Sequence	Length (aa)	Molecular Weight (Da)	Docking Energy (kcal/mol)
1	DFLDDGPG	8	821.75	−7.7
2	SDTGNFGF	8	829.80	−7.6
3	FGSLGNP	7	705.74	−7.5
4	PDAILP	6	600.71	−7.5
5	ADIDF	5	593.59	−7.4
6	FGDMDF	6	754.70	−7.4
7	DPDEGIRF	8	947.86	−7.3
8	PATPVF	6	611.74	−7.1
9	ASGDPWG	7	701.70	−7.1
10	PIDALP	6	600.71	−7.0
11	DAFDVP	6	643.72	−7.0
12	IDGAVFP	7	713.79	−7.0
13	GPSLKGGP	8	742.84	−6.9
14	IPDLPQP	7	770.89	−6.9
15	IPGELP	6	640.73	−6.9
16	PGSTPLP	7	701.79	−6.9
17	DPISSPLI	8	829.99	−6.9
18	EGDIF	5	607.57	−6.7
19	SPEGSGMP	8	786.80	−6.7
20	GLPDDF	6	676.71	−6.7
21	IDGPMP	6	642.76	−6.6
22	AGAGGSFPN	9	842.85	−6.5
23	GSGDLIP	7	689.76	−6.4
24	MVPDLP	6	658.80	−6.4
25	MSRLCAP	7	799.95	−6.3
26	IDPVGLP	7	700.80	−6.3
27	IDLFM	5	677.77	−6.2
28	EEGPCLLP	8	863.92	−6.2
29	LDCKPPL	7	790.97	−6.2
30	DGILLPGK	8	829.96	−6.1
31	IDGGLP	6	584.67	−6.1
32	IDPDFID	7	819.76	−6.1
33	MKMSLL	6	733.96	−5.9

## Data Availability

The original contributions presented in the study are included in thearticle/Supplementary Material, further inquiries can be directed to the corresponding authors.

## Supplementary Materials

The following supporting information can be downloaded at: https://www.mdpi.com/article/10.3390/foods15040673/s1, Figure S1: Umami receptor T1R1/T1R3 protein structure (A: T1R1 schematic, B: T1R3 schematic, C: 3D diagram of T1R1/T1R3 modeling results). (D) Ramachandran plot score of T1R1/T1R3 protein model. Table S1. Extraction yields (soluble solids, protein, and total sugar) of five single-enzyme.
