# Supplementary material for: Preparation and Identification of the Novel Umami Peptides from Sea Cucumber Viscera Hydrolysate
Source: Foods. 2026 Feb 12;15(4):673. doi: 10.3390/foods15040673 (PMC12939894; doi:10.3390/foods15040673)
Supplement: Supplementary file 1 [file foods-15-00673-s001.zip › foods-4120055-supplementary.pdf]

Supporting Information

Preparation and identification of the novel umami peptides from sea cucumber viscera hydrolysate

Xinmiao Ren [1†], Yiling Zhong [1†], Changyun Wang [2], Qingping Liang [1], Shuang Li [1], Rongqiang Chen [1], Dongyu Li [1], Changliang Zhu [1], Xiaodan Fu [1,*], Haijin Mou [1,*]

[1]College of Food Science and Engineering, Ocean University of China, Qingdao 266404, China;

[2]School of Medicine and Pharmacy, Ocean University of China, Qingdao 266003, China; changyun@ouc.edu.cn

*Corresponding author:

Xiaodan Fu

Email address: luna_9303@163.com

Haijin Mou

Email address: mousun@ouc.edu.cn

Contents of Supporting Information

Fugure S1.

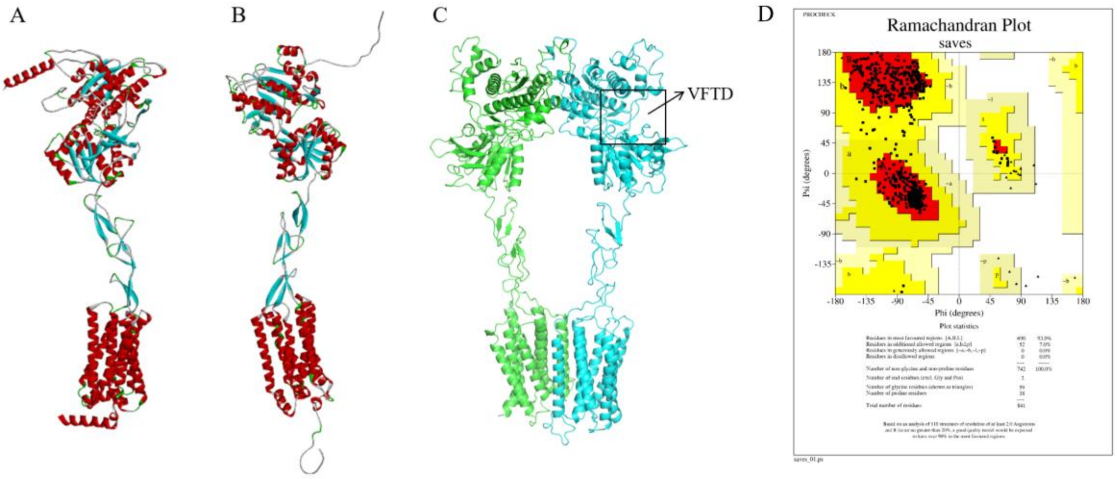

Figure S1. Umami receptor T1R1/T1R3 protein structure (A: T1R1 schematic, B: T1R3 schematic, C: 3D diagram of T1R1/T1R3 modeling results). (D) Ramachandran plot score of T1R1/T1R3 protein model.

Table S1.

Table S1. Extraction yields (soluble solids, protein, and total sugar) of five single-enzyme

Extraction rate(%) / Protease	Soluble solid substance	Protein	Total sugar
CK	15.65 ± 0.24[d]	5.82 ± 0.17[e]	5.68 ± 0.16[e]
Alcalase	49.36 ± 0.44[a]	50.75 ± 0.30[a]	48.94 ± 0.44[ab]
Neutrase	36.83 ± 0.48[c]	35.39 ± 0.48[c]	37.54 ± 1.41[c]
Subtilisin	45.03 ± 0.54[b]	46.06 ± 1.07[b]	37.58 ± 1.11[c]
Papain	30.42 ± 0.18[d]	22.46 ± 0.46[d]	51.81 ± 0.31[a]
Flavourzyme	48.90 ± 0.77[a]	49.77 ± 0.37[a]	46.78 ± 0.28[b]
